# Development and validation of a prognostic model for overall survival in small cell carcinoma of the esophagus

**DOI:** 10.3389/fonc.2025.1540691

**Published:** 2025-06-27

**Authors:** Xiaolei Yin, Xiaopeng Li, Lili Mi, Jiaojiao Hou, Fei Yin

**Affiliations:** ^1^ Department of Gastroenterology, Fourth Hospital of Hebei Medical University, Shijiazhuang, Hebei, China; ^2^ Medical Record Room, Fourth Hospital of Hebei Medical University, Shijiazhuang, Hebei, China

**Keywords:** prognostic model, small cell carcinoma of the esophagus, overall survival, nomogram, TNM, VASLG

## Abstract

**Background:**

Small cell carcinoma of the esophagus (SCCE) is an exceptionally rare subtype of esophageal carcinoma. Accurate survival prediction is challenging due to the lack of widely recognized prognostic models. This study aimed to construct and validate a prognostic model to predict overall survival (OS) in SCCE patients.

**Methods:**

A total of 491 SCCE patients were included from two sources: the Fourth Hospital of Hebei Medical University (n = 333, 2010–2020) and the SEER database (n = 158, 2000–2020). Patients were subsequently divided into training (n = 234), internal validation (n = 99), and external validation cohorts (n = 158). A prognostic model for OS was constructed using multivariable Cox regression in the training cohort, from which a relative survival risk score and nomogram were derived. Model performance was evaluated using the C-index, AUROCs, calibration curves, and decision curve analysis (DCA), and compared to TNM and VASLG staging systems. The Kaplan-Meier method estimated survival, and differences were assessed using the log-rank test.

**Results:**

Of the 491 patients, 314 (86.7%) were male, with a mean age of 66 years. Independent prognostic factors for OS, including TNM stage, surgery, and chemotherapy, were incorporated into a Cox model, termed the TSC model. The C-index for the TSC score in the training cohort (0.738; 95% CI, 0.615–0.845) was significantly higher than TNM (0.706; 95% CI, 0.507–0.796) and VASLG (0.657; 95% CI, 0.606–0.708). Likewise, AUROCs for the TSC score at 1, 3, and 5 years (0.713, 0.732, 0.816) outperformed both TNM (0.686, 0.682, 0.725) and VASLG (0.592, 0.609, 0.648). Moreover, calibration curves illustrated strong alignment between predicted and observed survival probabilities. DCA showed the nomogram provided superior net clinical benefits. High-risk patients had a median OS of 9.7 months, significantly shorter than 28.5 months for low-risk patients. These findings were validated in internal and external cohorts.

**Conclusions:**

To the best of our knowledge, the TSC model is the first fully validated prognostic model for SCCE, offering more accurate OS predictions than TNM and VASLG staging systems, and providing a valuable tool for personalized treatment.

## Introduction

As is well documented, small cell carcinoma of the esophagus (SCCE) is an exceptionally rare tumor originating in the esophagus, accounting for approximately 0.5–2.8% of all esophageal malignancies (1, 2). First reported by McKeown in 1952 (3), it is characterized by rapid progression, high metastatic potential, and an extremely poor prognosis. The lack of standardized treatment protocols for SCCE has led to the adoption of therapeutic strategies established for small cell lung cancer (SCLC). Nevertheless, the prognosis remains suboptimal, with approximately a 5-year survival rate of approximately 10% for patients with limited-stage disease and close to 0% for those with extensive-stage disease (4, 5). In addition, while the TNM system is extensively applied for esophageal cancer (EC) and the VALSG criteria are commonly utilized for SCLC, no specific criteria are available to predict the prognosis of SCCE and guide clinical treatment (6).

To date, nomogram-based prognostic models play a pivotal role in predicting cancer outcomes and informing treatment decisions. In various malignancies, including EC and SCLC, nomogram-based prognostic models that integrate clinical variables have demonstrated superior predictive accuracy compared to traditional TNM staging systems (7, 8). Although a few studies have attempted to develop prognostic models for SCCE, their limited sample sizes and the absence of independent external validation cohorts have significantly compromised the predictive value of these models (9, 10). Therefore, a robust nomogram-based prognostic model, with an adequate sample size and a standardized validation cohort, is urgently needed to accurately predict the prognosis of SCCE.

Conducting prospective randomized controlled trials is challenging due to the low incidence of SCCE. Consequently, retrospective studies focusing on this rare tumor are both necessary and valuable. This research aimed at developing and validating a prognostic model for overall survival that exceeds the predictive performance of traditional staging systems, thereby enhancing the accuracy of prognostic assessments for this rare tumor and providing more reliable support for clinical decision-making.

## Materials and methods

### Population and data

A total of 491 patients, including 333 from our institution and 158 from the SEER database, were ultimately included in this study. The patient data from our institution were collected between 2010-2020. The dataset “SEER Research Data, 17 Registries, Nov 2022 Sub (2000–2020)” was retrieved from the SEER database using SEER*Stat 8.4.3 software. Data were extracted based on the International Classification of Diseases for Oncology (ICD-O-3/WHO 2008) using tumor site code for the esophagus and the ICD-O-3 histology codes: 8002/3 (malignant tumor, small cell type) and 8041/3 (small cell carcinoma, NOS). Patients from the SEER database were restaged using the 8th edition of the AJCC system.

The inclusion criterion for the study cohort was a histologically confirmed diagnosis of SCCE. Exclusion criteria were as follows (1): the presence of a second primary malignancy, and (2) insufficient clinical or follow-up data. The study adhered to the Declaration of Helsinki and received approval from the Ethics Committee of the Fourth Hospital of Hebei Medical University (No. 2024KS033).

### Variables and follow-up

To ensure consistency with the clinical variables in the SEER database, this study analyzed 10 clinical characteristics, namely sex, age, tumor location, T stage, N stage, TNM stage, VALSG stage, surgery, radiotherapy, and chemotherapy. Due to the lack of a recognized staging system for SCCE, the present study used the TNM system for esophageal cancer and the VALSG system for SCLC. Pathological staging was applied to patients who underwent surgical intervention, while clinical staging was used for those who did not.

In this study, overall survival was defined as the time from diagnosis to death from any cause or to the last follow-up date for surviving patients, and was set as the primary endpoint. Follow-up data for SEER patients were acquired from the database. For patients at our center, follow-up was conducted via outpatient visits or telephone interviews until October 1, 2024.

### Model construction and validation

**Figure 1:**
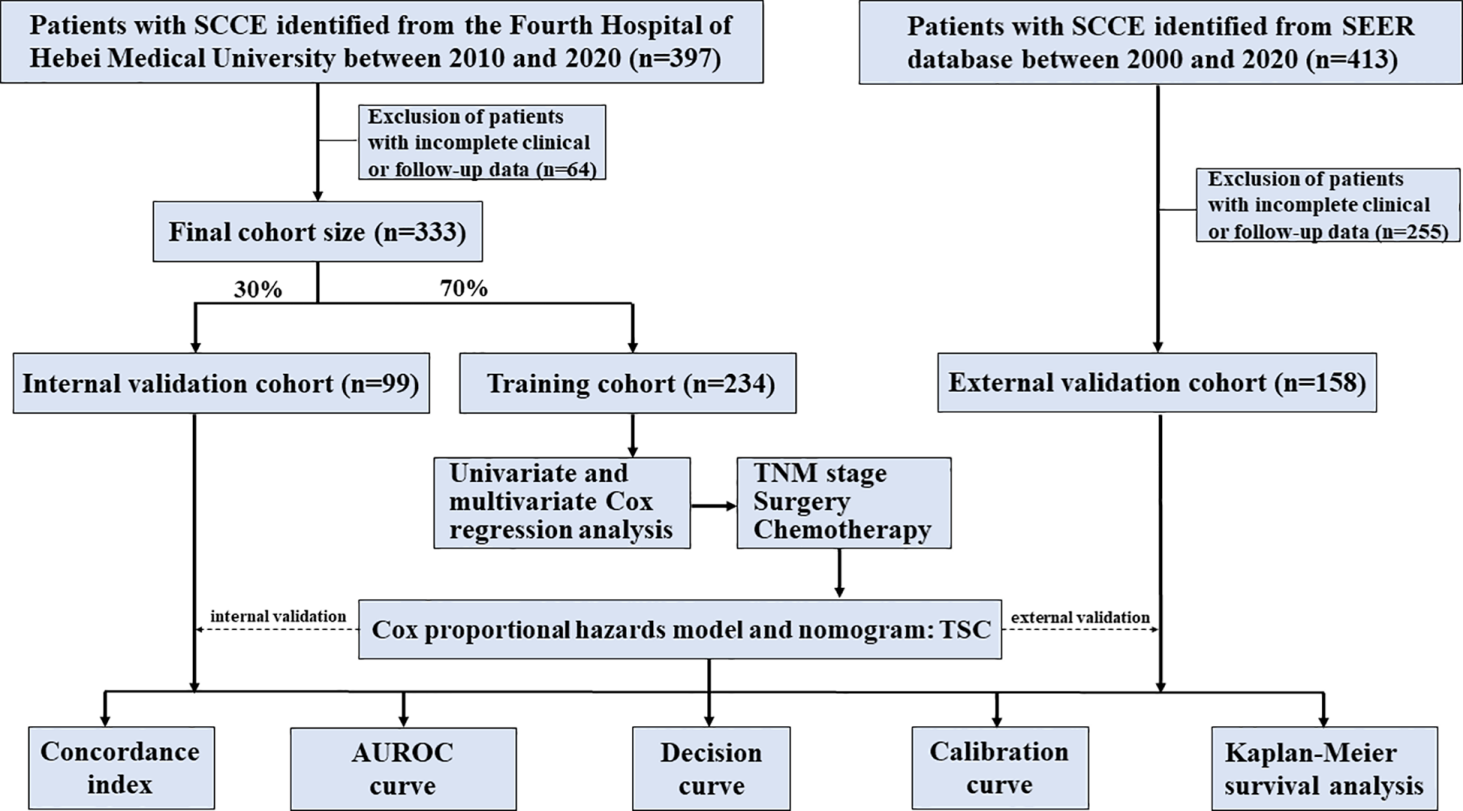
Flowchart of the prognostic model development process for SCCE.

The workflow of the modeling process is depicted in **Figure 1**. The dataset from our center was randomly divided into training and internal validation cohorts at a 7:3 ratio, with the SEER dataset serving as the external validation cohort. The training set data were initially analyzed using one-way analysis of variance to identify variables significantly linked to overall survival, and those with p-values <0.05 were subsequently included in the multifactorial analysis, with significant variables (p < 0.05) then incorporated into the final model.

We assessed the model’s discriminatory performance by calculating the C-index, AUROC, and DCA, and compared these metrics with those from other established prognostic systems, including TNM stage and VALSG stage. A nomogram derived from the finalized multivariate model was created, and calibration curves were generated to assess the match between predicted and actual OS outcomes.

### Statistical methods

Normal continuous variables are presented as mean ± SD, whereas non-normally distributed ones are categorized using the optimal cutoff from the survival R package. Categorical variables were expressed as frequencies and percentages. Kaplan-Meier curves and the Log-rank test were used for survival analysis, and Cox proportional hazards models were employed for performing univariate and multivariate analyses. The TSC score was computed as the weighted sum of selected variables, with weights derived from the β coefficients of the multivariate Cox model. The AUROCs and DCAs of the models were evaluated using the timeROC and ggDCA R packages (11, 12), respectively. Nomograms and calibration curves were created using the rms R package. All analyses were performed using R software (4.4.1), with statistical significance defined as a two-sided p-value < 0.05.

## Results

### Patient demographics and baseline characteristics

The baseline characteristics of patients are summarized in **Table 1**. The mean age was 65.58 ± 8.74 years in the training cohort, 63.13 ± 8.68 years in the internal validation cohort, and 67.55 ± 10.9 years in the external cohort, with corresponding sample sizes of 234, 99, and 158, respectively. Males predominated across all cohorts (training: 63.68%, internal: 61.62%, external: 65.82%). Middle esophageal tumors were most prevalent in the training (58.55%) and internal validation (60.61%) groups, while lower esophageal tumors were more frequent in the external cohort (60.76%). Most patients had tumors with shallow invasion depth (T1-T2), accounting for 63.68%, 61.62%, and 65.82% of the training, internal, and external cohorts, respectively. Conversely, tumors with deeper invasion depth (T3-T4) were more common in the external validation cohort (53.8%). Lymph node metastasis (N1-N3) distribution was as follows: training cohort: 73.93%, internal validation cohort: 73.74%, external cohort: 62.03%. N0 stage was more prevalent in the external cohort (37.97%) compared to the other cohorts (training: 26.07%, internal validation: 26.26%). Early-stage (I-II) was more common in the training (45.3%) and internal validation (45.45%) cohorts compared to the external cohort (33.54%), where late-stage (III-IV) was more prevalent (66.46%). Limited-stage disease constituted the majority across all cohorts (training: 71.37%, internal: 72.73%, external: 59.49%), whereas extensive-stage disease was more prevalent in the external validation cohort (40.51%) compared to the other cohorts (training: 28.63%, internal validation: 27.27%). Surgical treatment was more frequently performed in the training (37.61%) and internal validation (39.39%) cohorts compared to the external cohort (15.82%). As anticipated, chemotherapy was widely used across all cohorts (88.46%, 85.86%, and 78.48%), while radiation therapy was more frequent in the external validation cohort (55.7%) compared to the training (30.34%) and internal validation (34.34%) cohorts. Survival outcomes revealed high mortality rates in all cohorts, with the highest rate observed in the external validation cohort (89.87%), followed by the internal validation (87.88%) and training cohorts (81.62%).

**Table 1:** Baseline characteristics of the patients in the training and validation cohorts.

Characteristics	Training cohort (n=234)	Internal validation cohort (n=99)	External validation cohort (n=158)
Age, years	65.58 ± 8.74	63.13 ± 8.68	67.55 ± 10.9
Sex			
female	85 (36.32%)	38 (38.38%)	54 (34.18%)
male	149 (63.68%)	61 (61.62%)	104 (65.82%)
Tumor location			
upper	23 (9.83%)	10 (10.1%)	14 (8.86%)
middle	137 (58.55%)	60 (60.61%)	48 (30.38%)
lower	74 (31.62%)	29 (29.29%)	96 (60.76%)
T stage			
T1-T2	178 (76.07%)	67 (67.68%)	73 (46.2%)
T3-T4	56 (23.93%)	32 (32.32%)	85 (53.8%)
N stage			
N0	61 (26.07%)	26 (26.26%)	60 (37.97%)
N1-N3	173 (73.93%)	73 (73.74%)	98 (62.03%)
TNM stage			
I-II	106 (45.3%)	45 (45.45%)	53 (33.54%)
III-IV	128 (54.7%)	54 (54.55%)	105 (66.46%)
VALSG stage			
limited	167 (71.37%)	72 (72.73%)	94 (59.49%)
extensive	67 (28.63%)	27 (27.27%)	64 (40.51%)
Surgery			
yes	88 (37.61%)	39 (39.39%)	25 (15.82%)
no	146 (62.39%)	60 (60.61%)	133 (84.18%)
Radiation			
yes	71 (30.34%)	34 (34.34%)	88 (55.7%)
no/unknown*	163 (69.66%)	65 (65.66%)	70 (44.3%)
Chemotherapy			
yes	207 (88.46%)	85 (85.86%)	124 (78.48%)
no/unknown*	27 (11.54%)	14 (14.14%)	34 (21.52%)
Event			
alive	43 (18.38%)	12 (12.12%)	16 (10.13%)
dead	191 (81.62%)	87 (87.88%)	142 (89.87%)

AJCC, American Joint Committee on Cancer; VALSG, Veteran’s Administration Lung Cancer Study Group.

*Unknown option only available in External validation cohort.

### Development of the prognostic model

**Figure 2:**
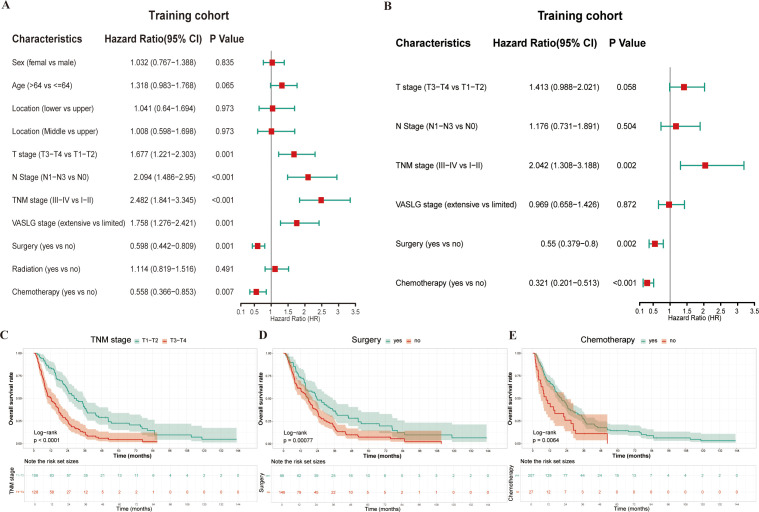
Prognostic analysis in the training cohort using univariate and multivariate Cox regression. **(A)** Forest plot of univariate Cox regression for variable selection; **(B)** Forest plot of multivariate Cox regression analysis; **(C–E)** Kaplan-Meier survival curves for the three significant variables (TNM stage, Surgery, and Chemotherapy) identified in multivariate analysis.

Univariate Cox regression identified six significant prognostic factors for OS in the training cohort (**Figure 2A**). These variables were subsequently included in the multivariate Cox model. Meanwhile, the multivariate analysis identified TNM stage (HR 2.042, 95% CI: 1.308–3.188, p = 0.002), surgery (HR 0.55, 95% CI: 0.379–0.8, p = 0.002), and chemotherapy (HR 0.321, 95% CI: 0.201–0.513, p < 0.001) were independent predictors of OS (**Figure 2B**). The Kaplan-Meier survival curves indicated that patients with advanced TNM stage (III–IV), those not undergoing surgery, and those not receiving chemotherapy experienced worse OS outcomes than their counterparts (**Figures 2C–E**). All differences in survival were statistically significant, supporting the prognostic value of these factors in predicting survival outcomes. Thus, TNM stage, surgery, and chemotherapy were used to construct the final TSC model, from which nomograms were developed for estimating relative survival risk scores and predicting absolute OS probabilities.

### TSC score and model validation

The relative risk score, derived from the finalized TSC model, was determined using the following method: TSC score = 0.903 × TNM stage (0, I-II; 1, III-IV) + (-0.544) × surgery (0, no; 1, yes) + (-1.071) × chemotherapy (0, no; 1, yes). The TSC score was calculated for all patients across different cohorts. Within the training set, the TSC score achieved a markedly higher C-index (0.738; 95% CI, 0.615–0.845) compared to both the TNM stage (0.706; 95% CI, 0.507–0.796) and the VASLG stage (0.647; 95% CI, 0.603–0.691) (**Table 2**). This trend was consistent across the validation sets, where the TSC score outperformed the other two variables. In the internal validation set, the C-index for the TSC score was 0.712 (95% CI, 0.508–0.856), while TNM and VASLG stages had C-indices of 0.680 (95% CI, 0.517–0.809) and 0.706 (95% CI, 0.480–0.862), respectively. In the external set, the TSC score yielded a C-index of 0.746 (95% CI, 0.614–0.844), surpassing the C-indices for TNM stage (0.631; 95% CI, 0.460–0.774) and VASLG stage (0.713; 95% CI, 0.591–0.765) (**Table 2**).

**Table 2:** C index of different models of overall survival in the training and validation cohorts.

Prognostic model	Training cohort	Internal validation cohort	External validation cohort
	C index (95% CI)	C index (95% CI)	C index (95% CI)
TSC score	0.738 (0.615-0.845)	0.712 (0.508-0.856)	0.746 (0.614-0.844)
TNM stage	0.706 (0.597-0.796)	0.680 (0.517-0.809)	0.631 (0.460-0.774)
VASLG stage	0.647 (0.603-0.691)	0.706 (0.480-0.862)	0.713 (0.591-0.765)

TSC, TNM stage-Surgery-Chemotherapy; TNM, the 8th edition of the American Joint Committee on Cancer tumor-node-metastasis staging system; VASLG, Veteran’s Administration Lung Cancer Study Group.

**Figure 3:**
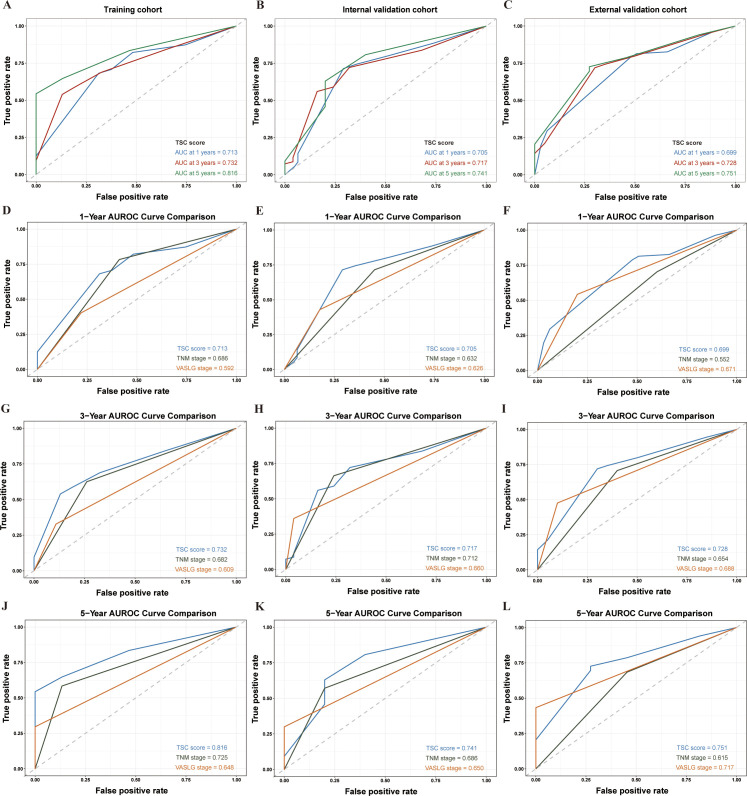
AUROC analysis of prognostic models in SCCE patients across different cohorts at 1, 3, and 5 years. **(A–C)** AUROC values for the TSC model in the Training, Internal Validation, and External Validation cohorts at 1, 3, and 5 years, respectively. **(D–F)** Comparison of AUROC for the TSC, TNM, and VASLG models at 1 year. **(G–I)** Comparison of AUROC for the three models at 3 years. **(J–L)** Comparison of AUROC for the three models at 5 years.

The AUROC values of the TSC score progressively increased over time in each cohort. The AUROC values in the training set were 0.782, 0.732, and 0.816 at 1, 3, and 5 years, respectively (**Figure 3A**). Likewise, they were 0.705, 0.717, and 0.741 in the internal validation set at the same time points (**Figure 3B**). Within the external validation set, the corresponding values were 0.699, 0.728, and 0.751 (**Figure 3C**). Indeed, the AUROC for the TSC score at 1, 3, and 5 years was consistently superior to the TNM and VASLG stages in each cohort. At 1, 3, and 5 years, the TSC score consistently showed higher AUROC values than the TNM and VASLG stages across all cohorts (training: 0.713 vs 0.686 vs 0.592; internal validation: 0.705 vs 0.632 vs 0.626; external validation: 0.699 vs 0.552 vs 0.571 for 1 year; training: 0.732 vs 0.682 vs 0.609; internal validation: 0.717 vs 0.712 vs 0.660; external validation: 0.728 vs 0.654 vs 0.688 for 3 years; training: 0.816 vs 0.725 vs 0.648; internal validation: 0.741 vs 0.686 vs 0.650; external validation: 0.751 vs 0.615 vs 0.717 for 5 years) (**Figures 3D–L**).

### Nomogram-based model performance and validation

**Figure 4:**
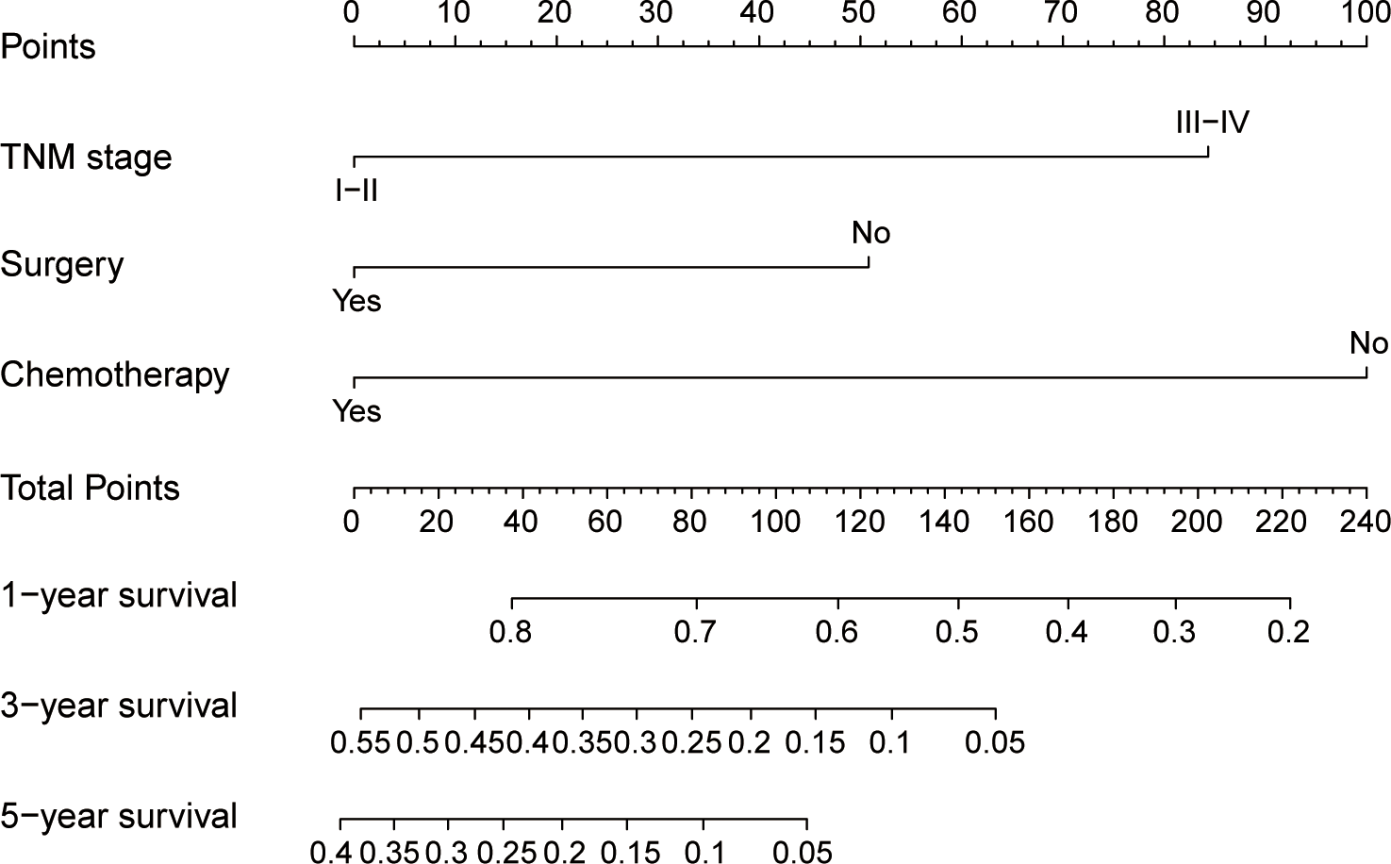
Nomogram for predicting 1-year, 3-year, and 5-year survival probabilities of SCCE. To estimate risk, calculate points for each variable by drawing a straight line from the patient’s variable value to the corresponding axis labeled “Points.” Sum the points for all variables and draw a straight line from the total points axis to the 1-year, 3-year, and 5-year OS axes to determine the survival probabilities.

**Figure 5:**
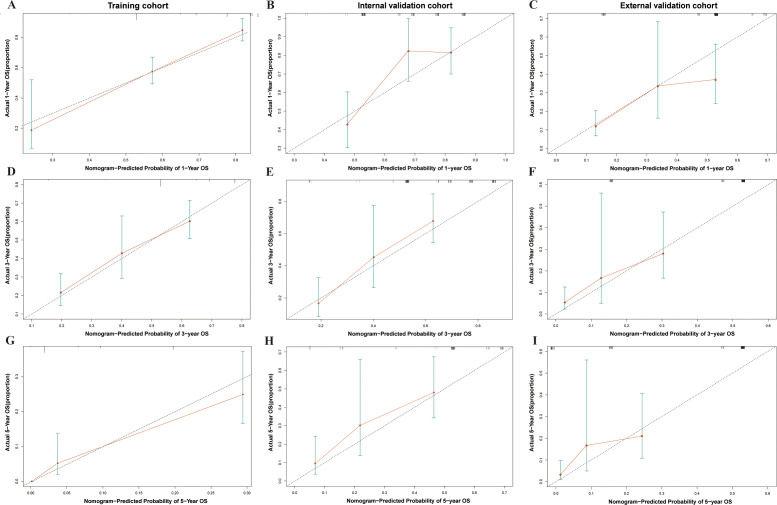
Calibration curves for 1-year, 3-year, and 5-year survival probability in SCCE patients across different cohorts. **(A–C)** Calibration curves for 1-year survival in the Training, Internal Validation, and External Validation cohorts. **(D–F)** Calibration curves for 3-year survival in the three cohorts. **(G–I)** Calibration curves for 5-year survival in the three cohorts.

**Figure 6:**
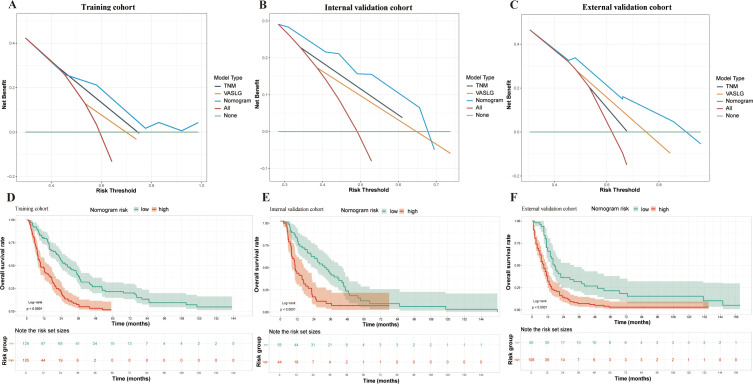
DCA and Kaplan-Meier survival curves in SCCE patients across different cohorts. **(A–C)** DCA for the three prognostic models (TSC, TNM, and VASLG) in the Training, Internal Validation, and External Validation cohorts. **(D–F)** Kaplan-Meier survival curves for patients stratified by Total Points from the nomogram in the three cohorts.

The nomogram (**Figure 4**), developed based on the TSC model, predicts the survival probabilities at 1, 3, and 5 years for individuals with SCCE. It incorporates TNM stage, surgery, and chemotherapy, which serve as key prognostic determinants, providing a clinically applicable risk prediction model. The calibration curves for the TSC model at 1, 3, and 5 years demonstrated strong consistency between predicted and observed survival probabilities across all cohorts (**Figures 5A–I**). At the same time, DCA revealed that the nomogram provided greater net clinical benefit compared to the TNM and VASLG staging systems (**Figures 6A–C**), demonstrating its superior clinical applicability.

The nomogram was constructed using the coefficients from the TSC model. Based on the cutoff values (2.919, 2.916 and 2.469) of the TSC score in each cohort, patients were stratified into low- and high-risk categories. The survival curves for the different risk groups showed significant differences in each cohort (**Figure 6D–F**). In the training cohort, high-risk patients had a median OS of 9.7 months, significantly shorter than the 28.5 months observed in the low-risk group. Comparable results were obtained in the internal (10.5 months vs 31.8 months) and external (9 months vs 17 months) validation cohorts. Taken together, the stratification results indicate that the newly developed nomogram can effectively distinguish survival outcomes for low- and high-risk patients.

## Discussion

In the present study, a prognostic TSC model was developed and validated to predict OS in SCCE patients. Of note, this model outperformed traditional TNM and VASLG staging systems in predictive accuracy, with higher C-index and AUROC values, indicating superior prognostic performance. The TSC score provides clinicians with a quantitative tool to assess survival risk in SCCE patients. Through the calculation of the TSC score, clinicians can classify patients into different risk categories (e.g., low and high risk), allowing for more tailored monitoring and treatment strategies. For high-risk patients, more intensive surveillance and therapeutic interventions can be considered. Additionally, the TSC nomogram, which predicts absolute overall survival probabilities over time, offers a practical and well-calibrated tool that can be integrated into routine clinical practice for more personalized patient management.

SCCE is a rare malignancy originating from the esophagus (13), and the majority of existing studies have focused on the more prevalent ESCC and EA, with limited prognostic studies conducted on small cell carcinoma (14–16). Although several earlier studies have attempted to identify effective molecular markers for prognostic prediction in SCCE, no markers with well-established clinical applications have been identified (17–19). Besides, different studies have yielded discrepant conclusions regarding markers such as Ki-67. Deng et al. (20) identified that high Ki-67 expression as a favorable independent prognostic marker in SCCE patients. However, Wang et al. (21) reported an inverse relationship. Several studies have also focused on the identification of clinical prognostic variables, and factors confirmed in these studies (22, 23), such as TNM staging, surgery, and chemotherapy, were similarly validated in our research. Nevertheless, radiotherapy has been more frequently recognized as an individual predictor of prognosis in studies using the SEER database (24, 25). This discrepancy may be ascribed to the higher proportion of American patients administered radiotherapy compared to Chinese patients. Specifically, only 31.53% of Chinese patients underwent radiotherapy in this study compared to 55.7% of American patients. At present, a standardized staging protocol for SCCE is lacking, and clinical practice primarily relies on the TNM staging system of esophageal carcinoma (26) or the VALSG staging system of SCLC (27). However, previous prognostic modeling studies (28) have primarily focused on TNM staging, with limited attention to VALSG staging. Herein, both systems were taken into account in the regression analysis. Unfortunately, the prognostic value of VALSG staging was not established, possibly due to the uneven distribution of patients with limited-stage versus extensive-stage disease (71.37% vs. 28.63%) in the training cohort. Future studies with larger sample sizes are warranted to accurately assess the prognostic relevance of VALSG staging.

Additionally, while existing SCCE prognostic models integrating these clinical variables have been developed, each has its own shortcomings. Chen et al. (9) identified a new molecular marker, BAR (ApoB/ApoA-1), and constructed a nomogram to predict SCC survival. However, the sample size used for model construction was small (61 individuals), and the nomogram lacked both calibration analysis and a critical external validation cohort, which may undermine the credibility and reliability of the prediction. Chen et al. (29) developed another model to predict survival in SCCE patients on the basis of nutrition- and inflammation-related metrics. However, the study faced issues such as a small sample size, lack of calibration analysis, and absence of external validation cohorts. Recently, although Qie et al. (10) used the SEER database to significantly increase the sample size, the authors acknowledged that clinical data on Asian populations were underrepresented, thereby limiting the applicability of the final prognostic model to Asian populations. Moreover, the study did not include an external validation cohort and exclusively performed internal validation, which somewhat limited the applicability of this model. Additionally, the clinical utility of the model was not assessed using DCA to calculate net benefits across various threshold probabilities. Besides the aforementioned limitations, the C-index reported in a recent study (30) was less than ideal, with a C-index of 0.659 for the training cohort. In contrast, this study aimed to minimize the limitations of previous studies by using different cohorts from China and the United States and employing multiple assessment and validation tools.

Initially, a standardized procedure was followed to develop a model for survival prediction in SCCE patients. To the best of our knowledge, this is the first study to incorporate both internal and external validation cohorts, utilizing a large and diverse sample to improve the robustness and generalizability of the prognostic model for this rare disease. The training cohort comprised 294 patients, with validation cohorts of 99 and 158 patients from China and the United States, respectively, ensuring broader applicability of the model. Additionally, this is the first prognostic modeling study to simultaneously compare the newly developed model with the two commonly used clinical models, TNM and VALSG, for SCCE. Regarding model consistency, classification accuracy, and risk assessment, the nomogram displayed superior predictive accuracy to both the TNM and VALSG staging systems. Noteworthily, a key strength of the nomogram is its inclusion of clinical variables that are vital for predicting survival, but are not considered by the TNM and VALSG staging systems. Notably, DCA showed that treatment decisions using the nomogram yielded greater net benefit compared to those relying on TNM stage, VALSG stage, or a one-size-fits-all approach. Furthermore, the model underwent thorough validation using multiple methods, including the C-index, AUROC, calibration curve, and DCA, which assessed its predictive accuracy and clinical utility at various time points. In conclusion, this model offers a reliable tool for accurately assessing the prognosis of SCCE patients, based on TNM staging, surgery, and chemotherapy, providing a reference for personalized treatment planning.

Radiotherapy was administered to 55.7% of patients in the SEER cohort, as reported in the Results section. In contrast, the proportion of patients receiving radiotherapy in our institutional training cohort was considerably lower. As the prognostic model was developed based on the training set, we followed a predefined stepwise variable selection strategy in which only factors demonstrating statistical significance in univariate Cox regression were retained for multivariate analysis. Radiotherapy did not meet this criterion and was therefore excluded from the final model. Nonetheless, radiotherapy remains an integral component of SCCE management, and its prognostic relevance may differ across populations due to differences in clinical practice patterns across countries. Further investigation in larger, treatment-stratified cohorts is warranted to better elucidate its potential role in outcome prediction.

Although our prognostic model demonstrated strong performance in predicting survival in SCCE, some limitations of this study cannot be overlooked. Firstly, the retrospective cohort study design inherently carries a risk of bias, underlining the need for prospective validation in future studies. Secondly, while the sample size is acceptable for a rare disease, it remains limited compared to studies on more prevalent cancers. This highlights the need for further validation through larger, multicenter cohorts to improve model stability and generalizability. Lastly, SCCE is a highly heterogeneous disease, and clinical factors alone may not fully capture prognostic risk. With advancements in sequencing technologies, molecular factors may offer additional insights into survival predictions. Therefore, single-cell sequencing of SCCE is currently being conducted to identify potential prognostic markers, further optimize existing models, and develop more reliable tools for survival risk prediction.

## Conclusion

Overall, this present study established and validated a prognostic TSC model for overall survival in SCCE. The developed model outperformed traditional staging systems (TNM, VASLG) in predictive accuracy, providing a robust tool for assessing survival risk in SCCE patients. By integrating key clinical variables, the model offers individualized risk stratification and dynamic survival predictions. Given the rarity of SCCE and the challenges associated with constructing prognostic models, future studies should focus on refining the model with larger cohorts and exploring molecular biomarkers to further improve its predictive performance.

## Data Availability

The raw data supporting the conclusions of this article will be made available by the authors, without undue reservation.
